# Smart Physiotherapy: Advancing Arm-Based Exercise Classification with PoseNet and Ensemble Models

**DOI:** 10.3390/s24196325

**Published:** 2024-09-29

**Authors:** Shahzad Hussain, Hafeez Ur Rehman Siddiqui, Adil Ali Saleem, Muhammad Amjad Raza, Josep Alemany Iturriaga, Álvaro Velarde-Sotres, Isabel De la Torre Díez, Sandra Dudley

**Affiliations:** 1 Institute of Computer Science, Khwaja Fareed University of Engineering and Information Technology, Abu Dhabi Road, Rahim Yar Khan 64200, Punjab, Pakistan; shahzad.hussain@kfueit.edu.pk (S.H.); adilalisaleem@gmail.com (A.A.S.); ch.amjadraza@gmail.com (M.A.R.); 2 Faculty of Computing, Riphah International University, 2 KM McDonald’s Lahore Multan Bypass Road, Sahiwal 5700, Punjab, Pakistan; 3 Facultad de Ciencias Sociales y Humanidades, Universidad Europea del Atlántico, Isabel Torres 21, 39011 Santander, Spain; josep.alemany@uneatlantico.es; 4 Departamento de Ciencias de Lenguaje, Educación y Comunicaciones, Universidad Internacional Iberoamericana Arecibo, Arecibo, PR 00613, USA; 5 Universidad de La Romana, Edificio G&G, C/ Héctor René Gil, Esquina C/ Francisco Castillo Marquez, La Romana 22000, Dominican Republic; 6 Facultad de Ciencias de la Salud, Universidad Europea del Atlántico, 39011 Santander, Spain; alvaro.velarde@uneatlantico.es; 7 Departamento de Salud, Universidad Internacional Iberoamericana, Campeche 24560, Mexico; 8 Faculdade de Ciências de Saúde, Universidade Internacional do Cuanza Bairro Kaluanda, Cuito EN 250, Bié, Angola; 9 Department of Signal Theory, Communications and Telematics Engineering, University of Valladolid, 47011 Valladolid, Spain; itordie@gmail.com; 10 Bioengineering Research Centre, School of Engineering, London South Bank University, 103 Borough Road, London SE1 0AA, UK; dudleyms@lsbu.ac.uk

**Keywords:** telephysiotherapy, PoseNet, exercise classification, machine learning, ensemble models, healthcare technology, Google MediaPipe

## Abstract

Telephysiotherapy has emerged as a vital solution for delivering remote healthcare, particularly in response to global challenges such as the COVID-19 pandemic. This study seeks to enhance telephysiotherapy by developing a system capable of accurately classifying physiotherapeutic exercises using PoseNet, a state-of-the-art pose estimation model. A dataset was collected from 49 participants (35 males, 14 females) performing seven distinct exercises, with twelve anatomical landmarks then extracted using the Google MediaPipe library. Each landmark was represented by four features, which were used for classification. The core challenge addressed in this research involves ensuring accurate and real-time exercise classification across diverse body morphologies and exercise types. Several tree-based classifiers, including Random Forest, Extra Tree Classifier, XGBoost, LightGBM, and Hist Gradient Boosting, were employed. Furthermore, two novel ensemble models called RandomLightHist Fusion and StackedXLightRF are proposed to enhance classification accuracy. The RandomLightHist Fusion model achieved superior accuracy of 99.6%, demonstrating the system’s robustness and effectiveness. This innovation offers a practical solution for providing real-time feedback in telephysiotherapy, with potential to improve patient outcomes through accurate monitoring and assessment of exercise performance.

## 1. Introduction

Telehealth encompasses the provision of healthcare services such as diagnosis, consultation, and education through electronic devices such as computers, laptops, and mobile phones [1]. This approach meets the evolving needs of an aging global population, which is expected to see the percentage of individuals aged 60 and above rise from 12% to 22% by 2050, nearly doubling from 2015 figures [2]. For elderly people with chronic diseases, the physical burden of traveling long distances to obtain healthcare presents a significant challenge. Telehealth offers a vital solution that facilitates remote access to healthcare services and effectively addresses these barriers. The COVID-19 pandemic has accelerated the adoption of telehealth, leading to improvements in service delivery [3]. Research shows that about 90% of patients and caregivers feel more secure with telehealth appointments compared to traditional in-person visits [4], highlighting its growing importance in improving healthcare accessibility and patient confidence.

Telehealth has also proven beneficial for remote physiotherapy, which focuses on diagnosing and treating impairments that hinder functional activities [5]. Physiotherapy often involves exercises that are crucial for patient rehabilitation. Consistent and accurate execution of these exercises allows therapists to adjust treatment as needed [6]. Traditional physical therapy involves direct interaction with the therapists who guide the patients to correct for exercise errors. However, the long duration and frequency of physiotherapy sessions make it challenging for patients to adhere to their regimens. Remote physical therapy via telehealth addresses these challenges by providing ongoing support and monitoring, thereby improving patient engagement and adherence to prescribed exercises. This paper presents a system that utilizes machine learning (ML) and PoseNet to appropriately categorize exercises. This novel technology contains an advanced method to accurately identify and classify different physical activities. The technology utilizes ML techniques and the PoseNet algorithm to examine the postures and movements of individuals while performing exercise routines. This comprehensive comprehension enables tailored feedback and assistance, thereby enhancing the efficacy of the rehabilitation process. The combination of ML with PoseNet in exercise categorization demonstrates the capacity of advanced technology to significantly improve the field of healthcare care, particularly in improving the precision and effectiveness of physiotherapeutic treatments. The contributions of this study are as follows:

- The dataset for this study was meticulously collected, comprising videos captured using a high-quality Hiievpu 2K Webcam with a superior CMOS 1/s image sensor. A total of 490 videos featuring subjects performing various exercises were included, with 350 featuring male subjects and 140 featuring female subjects.
- The Google MediaPipe library was employed to extract PoseNet features, focusing specifically on twelve key points related to exercises.
- This study introduces two innovative ensemble models, RandomLightHist Fusion and StackedXLightRF, combining the strengths of individual classifiers. These stacked models were trained on the standardized PoseNet features and evaluated on the test set.
- To validate the robustness and generalization capabilities of the models, K-fold cross-validation with five folds was employed.

## 2. Literature Review

An extensive examination of the pertinent literature reveals a wide range of approaches and advancements designed to improve the effectiveness of physical therapy exercises, ensure correct body alignment, and facilitate remote monitoring of exercise sessions. This section presents significant research work, each with distinct viewpoints and approaches which contribute to the overall understanding of the topic. The study conducted by [7] presented a method that aims to aid patients in their physiotherapy activities by promoting correct postures and reducing the likelihood of injuries. The Home Based Physiotherapy Exercise (HPTE) dataset was utilized, which consists of eight exercises specifically designed for the shoulder and knee. The system incorporates seven exercises with a range of modifications, with a focus on improving the user experience. Pose estimation techniques are used to identify important anatomical landmarks, which are then compared using dynamic time warping during training sessions. An intuitive desktop application with a three-dimensional avatar provides guidance to patients, assigning numerical values to exercises to provide feedback. The technology guarantees exceptional precision in determining and comparing position and orientation, establishing a threshold of precision 80%. In another approach, Ranasinghe et al. [8] aimed to revolutionize home-based exercises by merging Human–Computer Interaction (HCI) technologies with ML. Their system utilizes pose estimation, reinforcement learning, and a web-based application to monitor and deliver autonomous feedback on physiological movements. The level of exercise intensity is tailored according to the specific muscle strength of each individual, and complete reports with detailed information about the activity are produced. This system accommodates a wide range of users, including individuals engaged in therapy and fitness enthusiasts, improving their ability to achieve training goals in the comfort of their own homes.

In [9], the authors developed a virtual personal trainer system that uses Kinect cameras specifically for yoga and physiotherapy workouts. The Open Kinect driver acquires RGB and depth images, while the Kinect SDK retrieves real-time skeleton data. Users are guided by visual signals and audio instructions, which contribute to the development of a thorough training program. The installation of the Kinect sensor and software on a computer allows users to interact with the virtual personal trainer. The ePhysio system, developed by [10] is designed to remotely monitor musculoskeletal therapy. This is achieved through the use of Inertial Measurement Units (IMUs) and textile sensors. A wireless connection enables the transmission of data to a cloud system, facilitating the analysis of patient progress and the provision of feedback by healthcare professionals. The data stored in the cloud are utilized for continuous training and prediction of machine learning models. In [11], the authors employed PoseNet and K-Nearest Neighbors (KNN) in the field of yoga to achieve precise detection of yoga poses. Users are guided through a video presentation and provided with step-by-step directions to ensure proper posture. The model demonstrates a training accuracy of 93.56% and a performance accuracy of 98.51%.

In [12], the authors introduced BodyMTS, an innovative approach for categorizing strength and conditioning programs based on video recordings. Their study specifically examined issues related to storage and computing in military press activity. They tackled these challenges by utilizing body posture tracking to transform movies into time series data. BodyMTS demonstrates precision and robustness against interference by utilizing multivariate time series classifiers for training and prediction. BodyMTS shows comparable accuracy to deep learning algorithms while offering faster processing times and decreased model engineering needs. The system utilizes Human Pose Estimation and Multivariate Time Series Classification to offer feedback to physiotherapists, coaches, and patients. Despite the presence of intrinsic noise, the system attains an average accuracy of 87%, surpassing that of human experts. In [13], the authors presented an approach that utilizes video to categorize human movement by integrating Human Pose Estimation, Multivariate Time Series Classification, and Interpretation. Their objective was to provide input to physiotherapists, coaches, and rehabilitation patients. Instead of using sensor-based methods, their study employed video data captured by mobile cameras. The approach entails transforming videos into time series data using human posture estimation and subsequently training time series classifiers. Although there may be noise in the video capture and pose estimate, their system showed an incredible precision rate of 81%, highlighting the efficiency of the methodology in assessing CrossFit Workout Activities.

In [14], the authors developed an advanced deep learning model for monitoring and classifying human exercises. This was achieved through the use of posture estimation and a spatial–temporal graph convolutional network (ST-GCN). The model was trained using a dataset obtained from the internet. Using MediaPipe BlazePose, they extracted 33 essential points that describe the human body. The ST-GCN model, which has been assessed for accuracy in the top-1% and top-5% categories, has four different versions. Optimal performance was accomplished by dividing the spatial configuration into partitions and assigning weights to the importance of the edges that can be learned, leading to a top-1 accuracy of 41.75% and top-5 accuracy of 89.32%. In [15], the authors proposed a yoga posture coaching system that employs an interactive display and transfer learning. Their study aimed to avoid health problems caused by inappropriate yoga postures. It involved capturing fourteen poses using an RGB camera, with individuals executing each stance many times. Their study utilized data augmentation to evaluate six transfer learning models. Among these models, the TL-MobileNet-DA model was chosen due to its exceptional overall accuracy of 98.43%, sensitivity of 98.30%, specificity of 99.88%, and high Matthews correlation value of 0.9831. In [16], the authors developed an AI Gym Trainer posture estimation system that utilizes MediaPipe and OpenCV technology. Their solution effortlessly incorporates pretrained MediaPipe models to estimate posture, and flawlessly connects with OpenCV for image and video processing. The system records the user’s video input, identifies key points on the body using MediaPipe, then measures the angles between these points using OpenCV to analyze posture. The user receives prompt feedback and corrected recommendations, which were evaluated using bicep curls as a test scenario. The system is designed to perform well under different lighting conditions and to be resistant to obstructions. Its approach includes gathering and preprocessing data, training a pipeline using backpropagation, and evaluating the model. The evaluation criteria consisted of mean average precision (MAP) and mean per-joint position error (MPJPE), which were used to quantify the overall accuracy (visibility) as 90%.

A study on physical therapy was undertaken in [17] by using motion history photographs to analyze squats. The accuracy of movement was determined using motion image detection. Knee joint position and depth information was utilized to infer the location of the joint. Calculation of the MHG (Motion History Gradient) was performed to assess the presence of motion. The results were quantified by assessing the incidence of motion, expressed as recall and precision, which were 83% and 85%, respectively. Another study [18] aimed to classify nine upper extremity exercises using kinematic data captured by an IMU-based system. The exercises included standing row, arm external rotation, abduction 900, external rotation, bicep curl, forearm pronation/supination, wrist curls, lateral arm raise, front arm raise, and horizontal abduction. The study involved a cohort of fifty participants, each of whom conducted a series of exercises. Each exercise was repeated ten times, resulting in a total of 4500 trials. IMU sensors were placed on the right hand, forearm, upper arm, and chest. Multiple machine learning classifiers were trained to identify and categorize the exercises. The Random Forest classifier achieved the highest accuracy scores of 98.6% and 91.9% for detecting the triaxial join range. The study conducted by by [19] focused on real-time assessment of rehabilitation activities utilizing thermal imaging. A thermal camera was used to record real-time photographs of three compensatory movements (shrug, body sway, and body twist). A convolutional neural network (CNN) was trained specifically to identify compensating motions. The precision and recall criteria produced scores of 93% and 97%, respectively. In [20], the authors investigated the recognition of four rehabilitation exercises: the bird dog, cat camel, cobra stretch, and pelvic tilt. This investigation employed a solitary camera devoid of any further apparatus, to observe the patients. The OpenPose library was used to extract 25 essential points representing body joints. A total of thirteen and eight characteristics were chosen for the training of a long-short term memory (LSTM) deep neural network, attaining an accuracy of 97.50%. The study by [21] utilized a single camera to track human skeletal movements during performance of squat exercises. Their study used open-source MediaPipe technology to extract the essential parts of the human body. An evaluation model was constructed using human skeletal monitoring through MediaPipe, joint angle tracking, and signal filtering techniques (double exponential smoothing and mean square error). The mean squared error was employed as a threshold to distinguish squat exercise performance.

While the aforementioned studies contribute significantly to the domain of AI-assisted exercise monitoring, they are not without limitations. Several studies depend on particular datasets, which could limit the applicability of their suggested systems. The range of exercises included in datasets and the differences in body shapes across individuals may not completely capture the intricacy of real-life situations. Furthermore, several studies concentrated on specific workouts or body regions; thus, generalization of their results to a wider variety of physical activities may necessitate additional investigations. In addressing the limitations observed in existing studies, our proposed system aims for a more comprehensive and adaptable AI-assisted exercise monitoring solution. To this end, our proposed system leverages a diverse and extensive dataset encompassing a wide array of exercises performed by individuals with varying body types and fitness levels.

## 3. Materials and Methods

**Figure 1:**
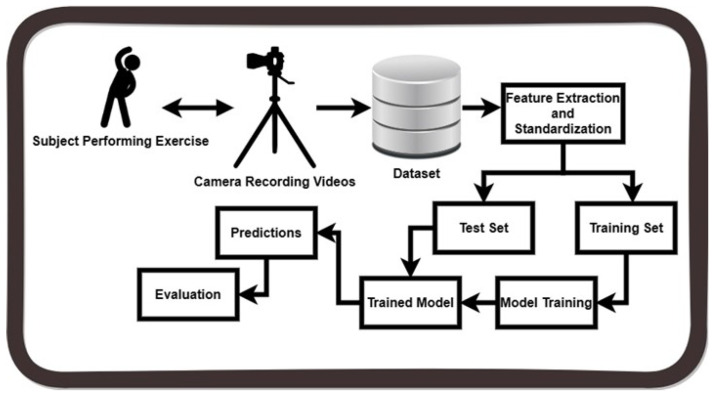
Diagram showing the proposed methodology for exercise classification.

The proposed methodology, depicted in Figure 1, presents a methodical strategy for classifying tasks. The first phase entails capturing video data using a webcam. These data are then sorted into several files labeled according to the type of exercise. These videos are used to extract PoseNet features, which are essential in the subsequent stages of analysis. After extracting the features, a standardization procedure is implemented to guarantee uniformity and compatibility for subsequent processing. The standardized features are divided into training and testing sets, which is a crucial stage in the machine learning (ML) and deep learning (DL) pipeline. The training set is fed into appropriate ML and DL classifiers, utilizing their ability to detect and understand intricate patterns in the data. The classifiers undergo fine-tuning and optimization using the training data, enabling them to efficiently learn and generalize exercise patterns. Afterwards, the classifiers are evaluated using the specified test set, based on which their performance is measured using different metrics such as the accuracy, precision, recall, and F1 score. These metrics offer a thorough assessment of the classification model’s efficacy in precisely classifying exercises. The proposed methodology follows a systematic and meticulous approach, incorporating computer vision techniques along with ML and DL methodologies to attain strong and dependable exercise categorization results.

### 3.1. Data Collection

This study was conducted with great attention to detail while showcasing a strong dedication to established ethical principles, and received formal approval from the Khwaja Fareed University of Engineering and Information Technology (KFUEIT) Ethics Committee. The Ethics Committee conducted a thorough evaluation of the ethical aspects related to the study, ensuring strict compliance with the principles specified in the Declaration of Helsinki. This primary emphasis on ethical norms highlights the significant value placed on protecting the welfare, rights, and privacy of all individuals participating in the research. The data collection process was carried out in the KFUEIT ICT building, with careful attention paid to ensuring compliance with ethical and research standards.

**Figure 2:**
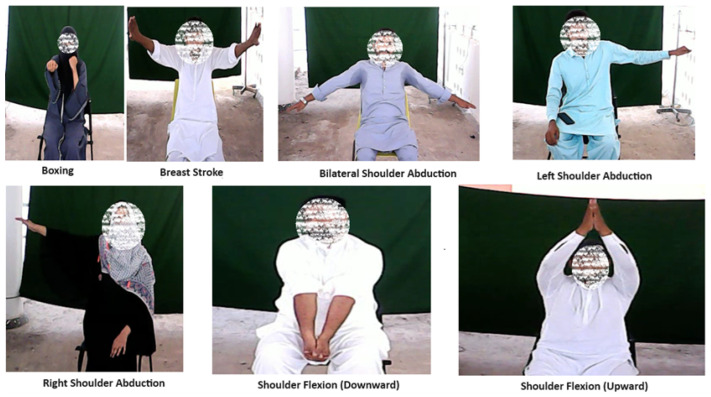
Subjects performing exercises.

The data collection phase involved a total of 49 subjects, comprising 34 males and 15 females falling within the age range of 21 to 29 years. Each participant engaged in a series of seven distinct exercises, including RSA, LSA, BSA, SFU, SFD, Breaststroke, and Boxing, as shown in Figure 2. The inclusion of these exercises was based on their relevance to shoulder joint rehabilitation [22,23]. Notably, the exercise duration for each task was standardized as 15 s, with a consistent distance of 2 m. Throughout the study, every subject performed each exercise precisely ten times, contributing to a comprehensive dataset that captures the nuances of the specific exercises in different participants.

Videos were recorded using a high-quality Hiievpu 2K Webcam [24], which is renowned for its exceptional features. This webcam incorporates a CMOS 1/s image sensor, allowing for the generation of high-definition images with an impressive resolution of 4 million pixels (2560 × 1440 p) [24,25]. Thanks to its seamless operation at a frame rate of 30 frames per second (fps), the advanced technological capabilities of this webcam facilitated the capture of videos with superior quality. During the exercise sessions, the subjects were provided with rest intervals between each set of exercises. The inclusion of rest periods served to mitigate potential fatigue and ensure that participants could perform each exercise with optimal effort and attention. This consideration for rest intervals not only aligns with established exercise protocols but also acknowledges the wellbeing of the subjects, promoting a safe and controlled environment for data collection.

### 3.2. Feature Extraction and Standardization

To ensure precise representation of the subtle temporal attributes of physical activity, frames were carefully extracted from the recorded videos at a rapid rate of 30 frames per second (fps). The intentional selection of this accelerated frame rate was crucial in accurately portraying the complexity and intricacy intrinsic in motion patterns. Next, the chosen frames were subjected to extensive processing utilizing the PoseNet feature extraction technique, which is a highly regarded algorithm recognized for its effectiveness in estimating the positions of individuals [26]. PoseNet conducts a thorough examination of the human body’s posture in every frame, identifying important anatomical landmarks such as joint positions, angles, and critical anatomical points. Recognizing these established landmarks provides significant insights into the spatial organization and dynamics of the human body when engaged in physical activity. In order to implement this procedure, our research used the Google MediaPipe library [27] as the main source to extract critical components of posture. MediaPipe is an innovation from Google that provides researchers with the ability to build perception pipelines. The MediaPipe library possess the capability to efficiently process and analyze a wide range of media inputs, including images and videos, and exhibits outstanding performance in a wide array of tasks, including hand movement tracking, body pose estimation, and face landmark recognition.

As depicted in Figure 2, the focus of this study is on exercises that predominantly involve arm movements. As a result, a methodical selection procedure was utilized to identify twelve crucial anatomical landmarks that are particularly pertinent to the arms: the right shoulder, right elbow, right wrist, right pinky, right index finger, right thumb, left shoulder, left elbow, left wrist, left pinky, left index finger, and left thumb. These points were selected with discerning and meticulous care from a larger population of 33 landmarks provided by the library. The selected landmarks allow for tracking the range of motion and coordination between key joints (shoulders, elbows, and wrists), which are central to exercises such as Right Shoulder Abduction (RSA), Left Shoulder Abduction (LSA), Bilateral Shoulder Abduction (BSA), Shoulder Flexion Upward (SFU), Shoulder Flexion Downward (SFD), Breaststroke, and Boxing. The X, Y, and Z coordinates of the key points and their related visibility ratings were obtained using the Cartesian coordinate system [28]. In order to maintain the accuracy and reliability of the data, this information was carefully maintained in a well organized file format known as Comma-Separated Values (CSV). The CSV file structure guarantees that each row corresponds to a distinct instance or frame in the video, while the columns indicate the X, Y, and Z coordinates of the posture markers along with their corresponding visibility ratings and labels.

After feature extraction, the next crucial step is standardization, which was performed using the widely recognized StandardScaler technique. This process ensures that all features are uniform and comparable by standardizing them with respect to their own mean and standard deviation. By removing the mean and then scaling each feature to the unit variance, the StandardScaler technique minimizes potential biases and variations that could arise from differences in the original measurement units. This standardized representation strengthens the consistency and reliability of the dataset, providing a solid foundation for subsequent machine learning and deep learning classification models.

### 3.3. Data Splitting

The dataset consisted of 490 videos showing subjects participating in workout activities, with 350 videos presenting male participants and 140 videos featuring female participants. The dataset was divided into two parts, with 70% used for training and 30% for testing, and was randomized prior to the split to ensure an even distribution and minimize potential biases during model evaluation. More precisely, 70% of the dataset, consisting of 343 videos (240 from males and 103 from females), was designated for training. The remaining 30%, consisting of 147 videos (110 from males and 37 from females), was set aside for testing. This deliberate division provides sufficient model training and rigorous evaluation on unfamiliar data, improving the ability of the exercise categorization system to generalize.

**Figure 3:**
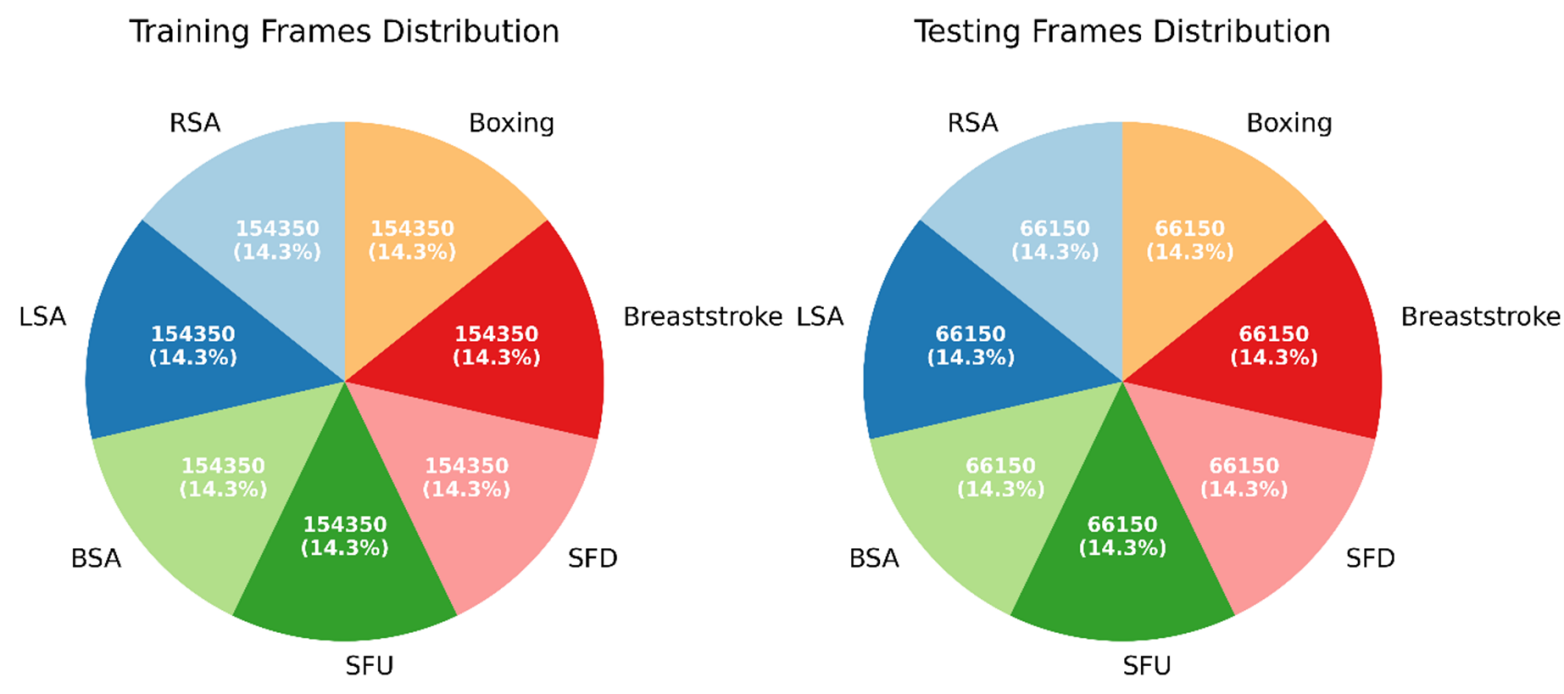
Frame distributions in the training and testing sets.

Every video in the dataset had a fixed duration of 15 s, helping to ensure uniformity in the temporal dimension. Frames were retrieved with great precision at a constant rate of 30 fps, capturing the dynamic subtleties of each exercise. The distribution of frame counts showed a precise balance within the dataset, as depicted in Figure 3. The equal distribution of frame counts among different exercise categories ensures a reliable basis for training models and conducting evaluations, demonstrating that our systematic methodology resulted in a well-rounded dataset for exercise categorization.

## 4. Results and Discussion

This section provides an extensive analysis and discussion of the results obtained from the experiments carried out in this study. The objective is to provide a comprehensive analysis of the results and to explain their significance within the framework of this research endeavor. In addition, a substantial discussion delves into the implications and significance of these results, contributing to a more nuanced understanding of the broader academic and practical implications of these findings.

### 4.1. Experimental Setup

The HP EliteBook x360 1040 G6 was used as the primary computing platform for our experimental investigations. The system was equipped with an Intel^®^ Core™ i5-8365U CPU @ 1.60 GHz (Intel Corporation, Santa Clara, CA, USA). which had a maximum clock speed of 1.90 GHz. As a result, the system demonstrated impressive processing capabilities. The CPU capability was enhanced by a significant 16.0 GB of RAM, providing enhanced multitasking and data management efficiency. The system utilized a 64-bit architecture and was equipped with Windows 11 Pro, demonstrating the smooth integration of advanced hardware and software components. This technical setup highlights the advanced capabilities utilized during the experimentation phase, guaranteeing a strong and adaptable computing environment.

### 4.2. Performance of Classifiers

In this study, we explicitly chose a set of tree-based machine learning classifiers for the classification tasks performed in our investigation: Random Forest (RF) [29], Extra Tree Classifier (ETC) [30], XGBoost [31], Light Gradient Boosting Machine (LGBM) [32], and Hist Gradient Boosting (HGB) [33]. These classifiers were chosen due to their robust ability to handle high-dimensional data, their flexibility in managing both linear and nonlinear relationships, and their proven effectiveness in multiclass classification tasks. Tree-based classifiers are particularly well suited for datasets with diverse features, as they can effectively manage feature interactions and are less prone to overfitting compared to other models. Additionally, these classifiers offer interpretability, scalability, and the ability to capture complex decision boundaries, making them ideal for the classification tasks in this investigation. Their ensemble nature also enhances generalization, leading to more accurate and reliable performance. In order to enhance the efficiency of these classifiers, we carried out a thorough adjustment of their hyperparameters. The hyperparameter values in Table 1 were meticulously selected using an exhaustive grid search, a systematic procedure aimed at discovering the optimal configuration for each classifier; this procedure guarantees that the classifiers are adjusted precisely to the subtleties of the dataset, thereby improving their ability to detect and apply the complicated patterns inherent in the exercise classification task.

Subsequently, the selected classifiers were extensively trained using the training set. After completing the training phase, the classifiers underwent extensive testing on the test set, which allowed for a strict assessment of their ability to generalize to new and unseen data. The findings of these classifications, which include measures of performance and evaluation outcomes, were thoroughly recorded and are provided in Table 2 with careful precision. This table shows how well and how differently each classifier performed in exercise classification.

The classification results in Table 2 exhibit the impressive performance of different tree-based machine learning classifiers. In particular, RF stands out as the best performing classifier, achieving an accuracy of 98.2%. This superior performance can be attributed to the ensemble nature of RF, which combines multiple decision trees to mitigate overfitting and enhance generalization. The ability of RF to effectively handle varied and intricate patterns within the dataset adds to its outstanding performance in exercise classification. Moreover, the ability of RF to capture the importance of features enables it to identify the essential properties that are pertinent to exercise categorization, leading to the development of a comprehensive and accurate model.

### 4.3. Performance of Stacked Classifiers

This paper presents an innovative ensemble model called RandomLightHist Fusion that combines three robust classifiers: HGB, LGBM, and RF. In addition, a secondary model called StackedXLightRF Fusion is built by merging XGBoost, LGBM, and RF. The hyperparameters used in these models are all in accordance with the configurations specified in Table 1, ensuring a consistent and rigorous experimental setup. The training phase consisted of providing the ensemble models with data from the training set, allowing them to effectively combine the strengths of each individual classifier. Afterwards, the recently developed stacked models were thoroughly evaluated on the test set, offering a detailed assessment of their combined performance in exercise categorization. Table 3 presents the classification scores and metrics obtained from this evaluation process, providing insight into the effectiveness of the proposed RandomLightHist Fusion and StackedXLightRF Fusion models.

The classification results displayed in Table 3 demonstrate the exceptional performance of the RandomLightHist and StackedXLightRF ensemble models introduced above. The RandomLightHist fusion algorithm demonstrates an exceptional accuracy of 99.6% along with remarkable precision, recall, and F1 score. This exceptional performance can be ascribed to the synergistic amalgamation of HGB, LGBM, and RF in this ensemble. The RandomLightHist ensemble’s capacity to utilize the advantages of the individual classifiers while minimizing their potential drawbacks leads to a very resilient and precise model. This model’s exceptional precision, recall, and F1-score demonstrate its ability to accurately classify exercise categories without any instances of false positives or false negatives. Similarly, the StackedXLightRF Fusion model attains a commendable accuracy of 99.2% with high precision, recall, and F1-score. The success of this outcome can be ascribed to the meticulous incorporation of XGBoost, LGBM, and RF into the ensemble. The stacking approach allows the component models to complement each other, compensating for their individual limitations and collectively enhancing the model’s overall predictive power.

### 4.4. K-Fold Cross-Validation Results

The proposed models were validated using K-fold cross-validation, a robust technique adopted in this study with a five-fold configuration. This method entails dividing the dataset into five separate folds; each fold takes a turn as the validation set, while the other folds together form the training set. This operation is iterated five times, guaranteeing that each fold is employed as the validation set precisely once. The extensive outcomes of this cross-validation process are thoroughly recorded in Table 4, offering a careful assessment of the models’ ability to generalize across various subsets of the dataset.

The cross-validation scores presented in Table 4 offer a thorough view of the performance and consistency of each classifier across various folds. The RF and ETC models have similar mean accuracy of 0.98, with RF displaying a slightly smaller standard deviation of 0.02. This suggests that RF had more stable and consistent performance over different folds. HGB, XGBoost, and LGBM provide average accuracy of 0.955, 0.95, and 0.95, respectively, with consistent standard deviations of 0.003 and 0.002. The RandomLightHist and StackedXLightRF ensemble models showcase notably higher mean accuracy of 0.997 and 0.99, respectively, both with standard deviations of 0.002. The ensemble models not only achieve high mean accuracy but also demonstrate low variability, underscoring their robustness and stability across different subsets of the dataset.

### 4.5. Comparison with Existing Studies

**Figure 4:**
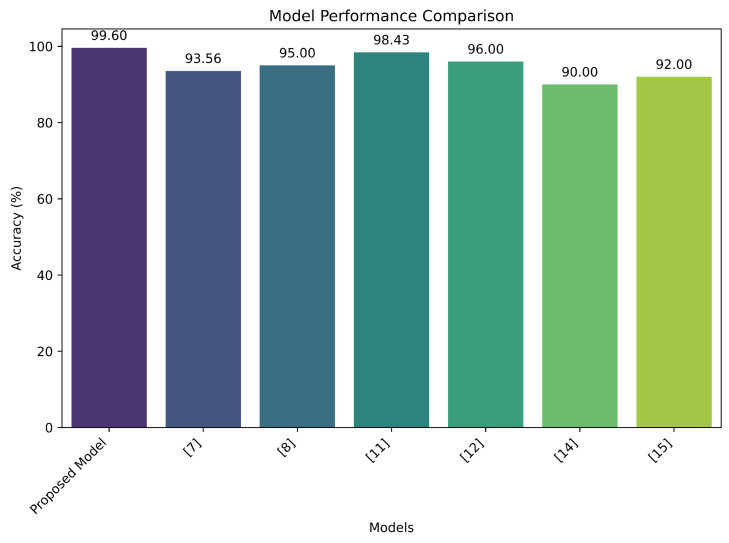
Comparison with existing studies.

This study proposes two novel ensemble models, RandomLightHist Fusion and StackedXLightRF, for real-time classification of exercises in telephysiotherapy. To evaluate the effectiveness of these models, a performance comparison was conducted with several existing methods from the literature. Figure 4 presents a comparative analysis of the accuracy of our proposed models alongside other prominent models discussed in the literature. As illustrated, the RandomLightHist Fusion model achieves an impressive accuracy of 99.6%, outperforming all other models included in the comparison. This high level of accuracy underscores the robustness of our ensemble approach in accurately classifying physiotherapeutic exercises. In contrast, the models from [7,11] achieve accuracy of 93.56% and 98.43%, respectively. While these models demonstrate strong performance, they fall short of the accuracy achieved by our RandomLightHist fusion model. The difference in performance highlights the potential of our ensemble approach to provide more precise and reliable feedback in telephysiotherapy settings. Additionally, the StackedXLightRF model shows competitive performance, with an accuracy of 99.2%, which is notably higher than several existing models, including those from [8,15]. This further emphasizes the efficacy of our models in enhancing the accuracy of exercise classification.

The superior accuracy of the proposed models, as depicted in Figure 4, demonstrates their potential to significantly improve real-time feedback in telephysiotherapy. Accurate exercise classification is crucial for effective remote monitoring and assessment, especially for individuals performing physiotherapeutic exercises from home. The ability of the proposed models to achieve high accuracy across diverse body morphologies and exercise types suggests that they can enhance patient outcomes by providing reliable and actionable feedback. While our models show promising results, there are some limitations to consider. The dataset used in this study included a limited number of participants and exercises, which may have affected the generalizability of the results. Future work could involve expanding the dataset to include a broader range of exercises and participants with varying body types and fitness levels. Additionally, incorporating real-time validation and testing in diverse environments could further validate the models’ performance.

### 4.6. Validation of Results

To ensure the reliability of our findings, MediaPipe’s performance was validated against the CVzone library using the same twelve anatomical landmarks: the right and left shoulders, elbows, wrists, pinkies, index fingers, and thumbs. Both libraries were used to extract features, then the accuracy of the classifiers generated from each set of features was compared. The classifiers were applied to a series of seven distinct exercises: RSA, LSA, BSA, SFU, SFD, Breaststroke, and Boxing. The results of this comparison are presented in Table 5, showing that MediaPipe consistently demonstrated slightly higher accuracy across all classifiers compared to CVzone. For instance, the RandomLightHist classifier achieved 99.6% accuracy using MediaPipe features, compared to 96.42% accuracy using CVzone features; similarly, the StackedXLightRF classifier showed 99.2% accuracy for MediaPipe and 97.84% for CVzone. Across all classifiers, MediaPipe outperformed CVzone in terms of accuracy, with the differences ranging from approximately 0.2% to 3.2%.

To statistically compare the performance of MediaPipe and CVzone across the classifiers, a paired sample t-test was conducted to assess whether the difference in accuracy between the two libraries was statistically significant. The null hypothesis (H0) states that there is no significant difference between the accuracy results of the classifiers using MediaPipe and CVzone, while the alternative hypothesis (H1) assumes that MediaPipe produces significantly higher accuracy than CVzone.

Mean precision for MediaPipe: 97.47%

Mean precision for CVzone: 96.66%

Mean difference: 0.81%

After performing the paired *t*-test, the resulting *p*-value was found to be below 0.05, indicating that the difference in classifier accuracy between MediaPipe and CVzone is statistically significant. Therefore, the null hypothesis can be rejected, and it can be concluded that the classifiers using features extracted by MediaPipe performed significantly better than those using features from CVzone.

### 4.7. Discussion

The comprehensive evaluation of the classifiers and ensemble models provides significant insights into their efficiency and stability for exercise classification. The RF classifier demonstrated robust performance, achieving an accuracy of 98.2% on the test set. This performance is further supported by the K-fold cross-validation results, which yielded a mean accuracy of 0.98 and a small standard deviation of 0.002, highlighting the classifier’s stability and effectiveness in managing multiclass classification problems. These results underscore the suitability of RF for exercise recognition due to its ability to handle diverse classes and its resilience against overfitting.

In comparison, the RandomLightHist fusion ensemble model exhibited superior performance, achieving exceptional accuracy of 99.6% on the test set. The model also had outstanding precision, recall, and F1 score metrics. The K-fold cross-validation results demonstrated high consistency, with a mean accuracy of 0.997 and a minimal standard deviation of 0.002. The impressive performance of the RandomLightHist model can be attributed to its integration of three powerful classifiers: Hist Gradient Boosting (HGB), LightGBM (LGBM), and RF. The synergistic effect of these models enhances gradient boosting capabilities and improves precision, while RF’s ensemble nature helps to mitigate overfitting and promotes generalization. The high precision of RandomLightHist, with no false positives, is particularly crucial for accurate exercise categorization, where precise classification is essential for effective feedback and assessment.

Regarding the impact of gender imbalance on model performance, separate testing of the model on male and female data revealed an accuracy of 99.4% for male data and 99.2% for female data, as shown in Table 6. This minimal performance disparity between genders indicates that the model performs consistently across different body types, maintaining its effectiveness regardless of gender. These results suggest that the gender imbalance in the sample does not significantly impact the model’s performance. The slight variations observed here are attributed to natural differences in body structure, and do not substantially affect the overall classification accuracy. This indicates that the model is robust and performs consistently across diverse body morphologies. The high accuracy achieved for both male and female data further highlights the model’s robustness and adaptability in real-world applications.

## 5. Conclusions

This study advances the field of telephysiotherapy by developing a novel system for accurate exercise classification using PoseNet, addressing key technical challenges such as real-time classification, adaptability to diverse body morphologies, and variations in exercise movements. We meticulously assembled a dataset from 49 participants who were recorded performing seven distinct exercises, from which we used twelve anatomical landmarks extracted using the Google MediaPipe library to ensure the generalizability and robustness of the proposed system. The introduction of the Random Forest, Extra Tree Classifier, XGBoost, Light Gradient Boosting Machine, and Hist Gradient Boosting tree-based classifiers provided a strong foundation for classification; however, it is the development of two ensemble models, RandomLightHist Fusion and StackedXLightRF, that sets this research apart. The RandomLightHist Fusion model achieved an impressive accuracy of 99.6%, significantly outperforming the individual classifiers and demonstrating the power of ensemble learning in exercise classification. The innovation in this research lies in its combination of advanced pose estimation with machine learning, which not only enhances classification accuracy but also facilitates real-time monitoring of exercise performance. This capability is particularly significant in telephysiotherapy, where the absence of physical presence makes accurate assessment and guidance critical to patient progress. By offering precise automated feedback on exercise execution, the proposed system has the potential to substantially improve remote physiotherapy services, reducing the burden on healthcare professionals while ensuring that patients receive consistent and high-quality care. Several promising directions for future work have been identified. These include exploring additional anatomical landmarks to improve the precision of exercise classification and body posture detection. Advanced deep learning techniques could be integrated to allow for a more sophisticated analysis of physical movements. The system could be extended to handle more complex exercises and larger, more diverse datasets of participants, enhancing its applicability in telephysiotherapy scenarios. Additionally, the inclusion of derived features such as the rate of change, movement frequency, and distance between points could lead to a more sophisticated model. This would provide further insights into exercise dynamics and improve the system’s interpretability. Other potential advances include the incorporation of motion analysis, joint angle estimation, and real-time correction suggestions to provide immediate feedback during therapy sessions. These improvements will be essential in making the system more adaptable and practical for clinical use, ultimately enhancing its effectiveness in telephysiotherapy.

## Figures and Tables

**Table 1:** Hyperparameters of the classifiers used in this study.

Classifier	Hyperparameters
HGB	max_iter = 100, random_state = 42
LGBM	default
XGBoost	n_estimators = 1000, learning_rate = 0.1, max_depth = 6
ETC	n_estimators = 100, max_depth = 200, random_state = 0
RF	random_state = 142, max_depth = 150, n_estimators = 150

**Table 2:** Classification results of the classifiers on the test set.

Classifier	Accuracy (%)	Precision	Recall	F1-Score
RF	98.2	0.98	0.98	0.98
ETC	98.01	0.98	0.98	0.98
HGB	95.3	0.95	0.95	0.95
XGBoost	95.1	0.95	0.95	0.95
LGBM	94.88	0.95	0.95	0.95

**Table 3:** Classification results of the stacked classifiers on the test set.

Classifier	Accuracy (%)	Precision	Recall	F1-Score
RandomLightHist	99.6	1.0	1.0	1.0
StackedXLightRF	99.2	0.99	0.99	0.99

**Table 4:** K-fold cross-validation results.

Classifier	Accuracy/Std
RF	0.98 ± 0.002
ETC	0.98 ± 0.005
HGB	0.955 ± 0.003
XGBoost	0.95 ± 0.002
LGBM	0.95 ± 0.002
RandomLightHist	0.997 ± 0.002
StackedXLightRF	0.99 ± 0.002

**Table 5:** Accuracy results of the classifiers using features extracted from the Mediapipe and CVzone libraries.

Classifier	Accuracy Using Features Extracted by Mediapipe (%)	Accuracy Using Features Extracted by CVzone (%)
RandomLightHist	99.6	96.42
StackedXLightRF	99.2	97.84
RF	98.2	96.37
ETC	98.01	96.97
HGB	95.3	95.76
XGBoost	95.1	95.7
LGBM	94.88	94.57

**Table 6:** Model performance metrics for male and female data.

Gender	Accuracy (%)	Precision (%)	Recall (%)	F1 Score (%)
Male	99.4	99.5	99.4	99.4
Female	99.2	99.3	99.2	99.2

## Data Availability

Data will be provided on suitable request.
